# Hemifacial Spasm due to Compression of the Posterior Inferior Cerebellar Artery Aneurysm Treated with Botulinum Toxin Type-A: A Case Report

**DOI:** 10.1155/2012/132594

**Published:** 2012-07-16

**Authors:** Azize Esra Gürsoy, Gülsen Babacan Yildiz, Adam Mehmet Gülhan, Mehmet Kolukisa

**Affiliations:** ^1^Department of Neurology, Medical Faculty, Bezmialem Vakif University, Istanbul, Vatan Caddesi 34093 Fatih, Turkey; ^2^Neurology Department, Sentara Health System, Norfolka, VA 23502, USA

## Abstract

A 79-year-old female presented with five months history of progressive involuntary twitching movement on left face. Brain MR imaging revealed a heterogeneous T2 hyperintense lesion at left cerebellopontine angle. CT angiography showed a partially thrombosed saccular aneurysm of left PICA (posterior inferior cerebellar artery). The patient was treated with Botulinum toxin type A and almost total relief of symptoms was noticed during one month followup. Botulinium toxin injection is an effective symptomatic treatment option in nonsurgical secondary hemifacial spasm (HFS) cases.

## 1. Introduction

Hemifacial spasm (HFS) is a peripherally induced movement disorder characterized by involuntary tonic and clonic contractions of facial muscles innervated by ipsilateral facial nerve [1]. Most cases of primary HFS are attributed to an aberrant artery (anterior inferior cerebellar, posterior cerebellar, or vertebral) compressing CN VII at the root exit zone (REZ) [2]. The facial nerve compression is thought to lead to ephaptic transmission and to hyperactivity of the facial nucleus, resulting in the involuntary facial movements [3, 4]. Secondary causes of HFS are rare and composed of 0.3–5.1% of all cases [1, 5]. Cerebellopontine angle tumors, arteriovenous malformations, and less commonly aneurysms are mentioned among causes of secondary HFS [5, 6], which are generally treated with surgical interventions. Here, we presented a patient admitted to our clinic with involuntary contractions on left face and diagnosed as HFS secondary to saccular aneurysm of PICA. The patient was not a surgical candidate, and treated with Botulinum toxin type-A with successful symptomatic relief.

## 2. A Case Report

**Figure 1:**
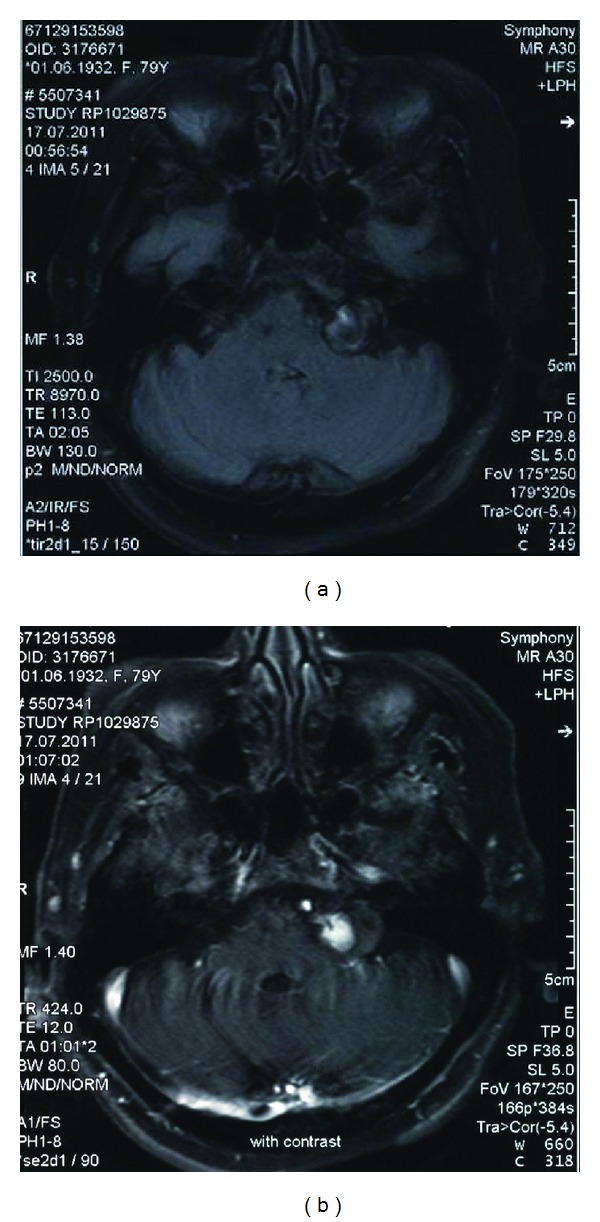
(a) and (b) Axial flair and axial postcontrat T1 sequences of cranial magnetic resonance imaging show a lesion with heterogeneous intensity with contrast enhancement.

**Figure 2:**
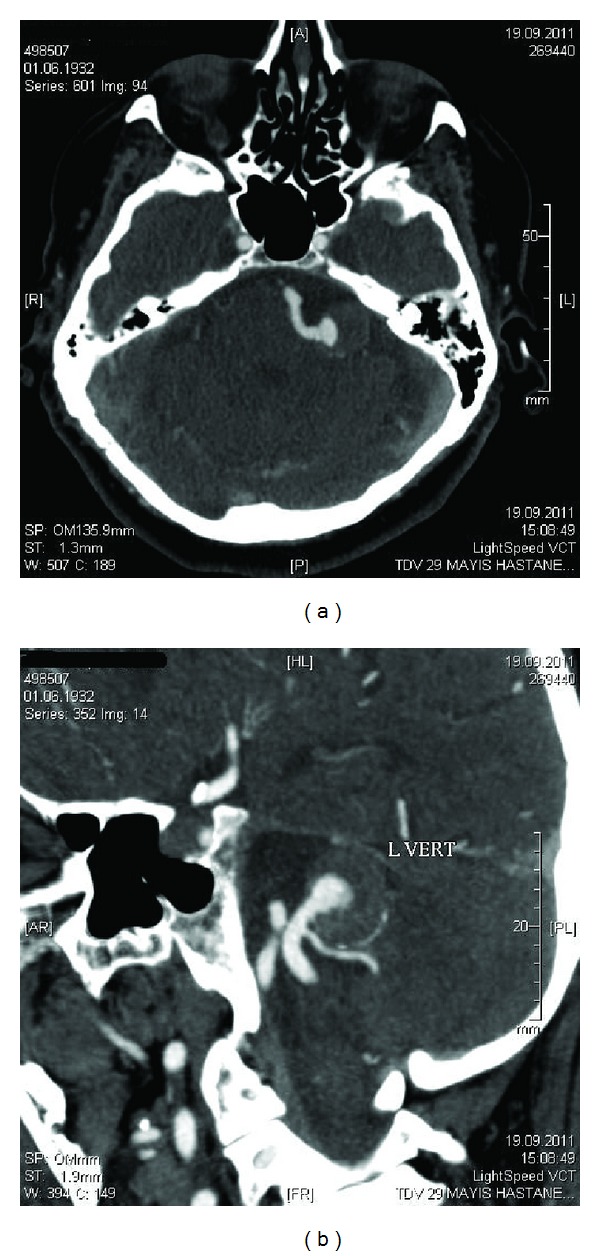
(a) and (b) CT angiography shows partially thrombosed saccular aneurysm with calcified walls, emerging from left PICA, just distal to its origin from vertebral artery on axial and saggital plane.

A 79-year-old female patient presented with involuntary muscular contractions of left face. Symptoms started five months ago, initially affecting left eyelids then involved ipsilateral lower facial muscles with gradually increased intensity and frequency of spasms during last three months. Past medical history was negative except hypertension. Physical examination did not reveal abnormality other than HFS which was severe at level 4 according to Jankovic rating scale (0 = no spasm, 4 = severe, incapacitating spasm) [7]. The patient had no complaints of hearing loss. Cranial MRI imaging showed a lesion with heterogeneous intensity at left cerebellopontine angle on flair sequence which enhanced contrast (Figures 1(a) and 1(b)). Additionally, flair and T2-weighted magnetic resonance images showed nonspecific hyperintense lesions of bilateral white matter, basal ganglia and thalamus and increased prominence of cerebral sulci. Following, CT angiography showed partially thrombosed saccular aneurysm in size of 22 × 18 mm with calcified walls, emerging from left PICA, just distal to its origin from vertebral artery (Figures 2(a) and 2(b)). Based on consultation with neurosurgery, surgical intervention was not considered due to the patient's old age and thrombosed feature of aneurysm. We decided to treat HFS with Botulinum toxin type-A. A total of 22.5 units of Botulinum toxin type-A (Botox) is injected into left orbicularis oculi, zygomaticus minus, zygomaticus majus, and mentalis muscles. The patient was reevaluated one month after the Botulinum Toxin application and almost complete improvement of HFS was noticed. There was no adverse reaction or complication.

## 3. Discussion

HFS is generally seen in population between age of 40–79. It occurs more commonly in women (2 : 1) with an overall prevalence of about 10/100 000. Primary HFS is mostly attributed to vascular loops compressing the seventh cranial nerve at its exit zone from brainstem [1, 8, 9]. Secondary HFS may arise from facial nerve damage produced by tumors, demyelinating disorders, arteriovenous malformations, trauma, and aneurysms. Han et al. reviewed 1642 cases of HFS and found nine of them (0.5%) were secondary to causative structural lesions, including seven cerebellopontine angle tumors, one arteriovenous malformation, and one developmental venous anomaly [6]. Colosimo et al. compared differences in the demographic and clinical features between primary and secondary HFS. They found that in most patients (65%) with primary HFS involuntary contractions started in the periocular muscles and then spread somatotopically to the neighboring facial muscles. Conversely, in most patients (72%) with secondary HFS, contractions simultaneously involved the upper and lower facial muscles [10]. Anatomical data suggest that the facial nerve motor fibers are topographically organized along their courses into pons and, probably, at the root exit zone [11]. The fibers become more diffusely arranged at distal levels of the facial nerve. In Colosimo's report, secondary HFS group included high percentage of patients with prior peripheral facial palsy [10]. We think that this might be a reason of high rate of simultaneous involvement of upper and lower facial muscles in their secondary HFS group. In our patient, HFS was secondary to PICA aneurysm, nevertheless, facial spasms started in upper face first then spread to lower facial muscles, as typically seen in primary HFS. This was likely because of the location of thrombosed PICA aneurysm and its similar effect on facial nerve REZ as typically seen in primary HFS. Reported cases of HFS caused by PICA aneurysm are rare in literature [12–15]. Indeed posterior inferior cerebellar artery (PICA) aneurysms are rare conditions, accounting for 0.5–3.0% of all intracranial aneurysms [16, 17]. Surgical clipping or endovascular coiling is the choices of treatment when the risk of rupture is considered to exceed the therapeutic risks. The indications for surgical treatment of PICA aneurysms in the presence of a hemifacial spasm remain controversial. Reported cases in the literature were commonly treated with microvascular decompression or endovascular embolisation. International Study of Unruptured Intracranial Aneurysms Investigators reported that age is an important factor in overall surgical outcome, with a substanial increase in risk for those about 50 years and older, which rises substantially after age 60–70 years. Other predictors of poor surgical outcome were reported in the same study as large aneurysmal size, location in the posterior circulation, history of ischaemic cerebrovascular disease, and presence of aneurysmal symptoms other than rupture [18]. Our patient was not a good candidate for open surgery and endovascular embolisation because of patient's age, location of the aneurysm, and symptomatic mass effect of the partially thrombosed aneurysm. Symptomatic treatment of HFS with Botulinum toxin is a safe and effective option [19–22]. We used Botulinum toxin type-A in our case to treat HFS caused by thrombosed PICA aneurysm and symptoms were relieved almost completely with Botulinum Toxin treatment.

Because secondary HFS may present clinically similar to primary HFS, radiological investigation is important even in patients present with typical HFS symptoms without additional neurological symptoms and signs. Botulinum toxin type-A is an effective symptomatic treatment option of secondary HFS.
